# Dynamics, circuit design, feedback control of a new hyperchaotic system and its application in audio encryption

**DOI:** 10.1038/s41598-023-46161-5

**Published:** 2023-11-08

**Authors:** ShiMing Fu, XueFeng Cheng, Juan Liu

**Affiliations:** 1 School of Artificial Intelligence, Chongqing University of Education, Chongqing, 400065 China; 2 School of Big Data and Information Industry, Chongqing City Management College, Chongqing, 401331 China; 3 Chongqing University, Chongqing, 400044 China; 4 Chongqing Institute of Green and Intelligent Technology, Chinese Academy of Sciences, Chongqing, 400714 China; 5 Chongqing College, University of Chinese Academy of Sciences, Chongqing, 400722 China

**Keywords:** Electrical and electronic engineering, Nonlinear phenomena

## Abstract

In this study, a 4D hyperchaotic system is constructed based on the foundation of a 3D Lü chaotic system. The newly devised hyperchaotic system possesses a sole equilibrium point, showcasing a simplified system structure that reduces complexity. This simplification offers a clearer opportunity for in-depth analysis of dynamic behaviors in the realm of scientific research. The proposed hyperchaotic system undergoes an in-depth examination of its dynamical characteristics, including chaotic attractors, equilibrium point stability, Lyapunov exponents’ spectrum, and bifurcation diagram. Numerical analysis results reveal that the attractor of this hyperchaotic system exhibits highly complex, non-periodic, and fractal structural dynamics. Its motion demonstrates extreme sensitivity and randomness, even within a wide range of variations in parameter *d*, affirming its hyperchaotic properties with two positive Lyapunov exponents. Hyperchaotic bifurcation diagrams typically exhibit highly intricate structures, such as fractals, branches, and period doubling characteristics, signifying that even minor parameter adjustments can lead to significant changes in system behavior, presenting diversity and unpredictability. Subsequently, to further investigate the practical utility of this hyperchaotic system, a linear feedback control strategy is implemented. Through linear feedback control, the hyperchaotic system is stabilized at its unique equilibrium point. Experimental validation is conducted using both computer software simulation Matlab, electronic circuit simulation Multisim, and embedded hardware STM32. The results of these experiments consistently align, providing theoretical support for the application of this hyperchaotic system in practical domains. Finally, leveraging the hyperchaotic keys generated by this hyperchaotic system, audio encryption is achieved using a cross-XOR algorithm, which is then realized on the embedded hardware platform STM32. The results show that the audio encryption scheme based on the hyperchaotic system is feasible, and the method is simple to implement, has nonlinear characteristics and certain algorithm complexity, which can be applied to audio encryption, image encryption, video encryption, and more.

## Introduction

In today's interconnected and data-driven world, ensuring the security and confidentiality of information is of paramount importance. The exponential growth in digital communication, online transactions, and data sharing has led to an unprecedented need for robust data protection mechanisms. This demand for enhanced security is fueled by the ever-present risk of unauthorized access, cyberattacks, and data breaches. As a result, encryption has emerged as a fundamental and indispensable tool in the realm of cybersecurity.

Encryption is the process of converting plaintext information into ciphertext, rendering it unintelligible to unauthorized parties. It serves as a powerful shield that safeguards sensitive data from prying eyes and potential adversaries. Over the years, encryption techniques have evolved to keep pace with advancing technology and increasingly sophisticated threats. While classical encryption methods, such as symmetric and asymmetric cryptography, have proven effective, the emergence of hyperchaotic systems introduces a new frontier in data security.

This paper embarks on an exploration of hyperchaotic systems and their profound implications for encryption. Chaotic systems, known for their deterministic randomness and sensitivity to initial conditions, have long captured the imagination of researchers. Hyperchaotic systems, a subset of chaotic systems, exhibit even greater complexity and unpredictability, making them a compelling candidate for enhancing data security.

Over the past 40 years, hyperchaotic systems^1–6^ have maintained a steady pace since the first such system was reported by Rŏssler. Recently, a large number of different hyperchaotic systems^7–28^ have been proposed, and due to their more complex dynamics, they have found widespread applications in electronics, communications, information processing, neuroscience, and other fields, including image encryption^29–41^, audio encryption^42–46^, video encryption^47–53^, and secure communication^54–63^.

While electronic circuit simulation software^2,3,27,28^ and embedded hardware (DSP^13–18^, FPGA^19–25^) have been widely used for simulating and controlling hyperchaotic systems, the application of embedded hardware STM32 has been limited. The STM32 offers high performance, low cost, low power consumption, real-time capabilities, digital signal processing, and connectivity, making it very popular in various industries such as industrial control, communications, and the Internet of Things.

Some researchers have delved deeply into the use of pseudo-random number generators based on hyperchaotic systems to generate encryption keys^29–46^. Hyperchaotic systems are characterized by their high nonlinearity and randomness, making them an ideal choice for generating strong cryptographic keys. These keys can be used to perform the encryption and decryption of audio data. In this process, the keys generated by hyper-chaotic systems serve as seeds for mathematical operations on audio data, producing ciphertext to obfuscate the audio signal. The critical aspect of this method is that attackers find it extremely challenging to predict the keys generated by hyperchaotic systems, making decryption a formidable task.

Furthermore, researchers have explored parameterization methods for hyperchaotic systems to further enhance the security and performance of encryption algorithms^2,7,8^. This includes adjusting the initial conditions and parameters of hyperchaotic systems to generate more complex and random chaotic sequences. By carefully selecting parameters, the encryption strength of audio data can be increased, making it even more resistant to decryption. This parameterization approach provides greater flexibility and security for audio encryption.

Another crucial application area is the integration of hyperchaotic systems into embedded hardware to achieve real-time audio encryption and decryption^13–25^. This is particularly important for applications that require rapid response and high security, such as communication systems, audio storage devices, and speech recognition systems. By implementing hyperchaotic systems in embedded hardware, encryption and decryption operations can be performed directly on the device itself, without relying on external computational resources. This capability for real-time encryption is vital for safeguarding audio communication and data storage, especially in sensitive information transmission and storage scenarios.

Therefore, hyperchaotic systems have found significant applications in the field of audio encryption. They offer a highly secure and high-performance encryption solution through the generation of complex keys, parameterization, and integration into embedded hardware. These efforts will contribute to ensuring the confidentiality and integrity of audio data and provide robust security and privacy protection across various domains.

In this paper, a new 4D hyperchaotic system is proposed which based on the 3D Lü chaotic system^64^, and investigate its dynamic properties through analysis of the chaotic attractor, the stability of equilibrium points, the Lyapunov exponents’ spectrum, and the bifurcation diagram. Stabilizing the hyperchaotic system to its equilibrium point is achieved using a linear feedback control method. Subsequently, the phase portraits of the system and the trajectories of the controlled variables are realized using both electronic circuit simulation software Multisim and embedded hardware STM32. In order to demonstrate the advantages of hyperchaotic systems in data encryption, this paper designs a cross-XOR operation encryption algorithm based on hyperchaotic sequence, and its application in audio encryption is implemented by embedded hardware STM32.

## A new hyperchaotic system

In this work, a new 4D hyperchaotic system described by:

1 $$ \left\{ \begin{gathered}   \frac{{{\text{dx}}}}{{{\text{dt}}}}\, =\, {\text{a}}\left( {{\text{y}} - {\text{x}}} \right){\text{ + w}} \hfill \\   \frac{{{\text{dy}}}}{{{\text{dt}}}}\, =\, {\text{cy}} - 10{\text{xz}} \hfill \\   \frac{{{\text{dz}}}}{{{\text{dt}}}}\, =\,  - {\text{bz + }}10{\text{xy}} \hfill \\   \frac{{{\text{dw}}}}{{{\text{dt}}}}\, =\, {\text{dy + x}}^{2}  \hfill \\  \end{gathered}  \right.. $$

where *x*, *y*, *z* and *w* are driving variables, and $\frac{{\text{dx}}}{{\text{dt}}}$,$\frac{\text{dy}}{{\text{dt}}}$,$\frac{\text{dz}}{{\text{dt}}}$,$\frac{\text{dw}}{{\text{dt}}}$ are derivatives of *x*, *y*, *z*, *w* respectively. The system's parameters are set as *a* = 35, *b* = 3, *c* = 12, with the variable parameter *d* > 0. Let the system (1) equals zero, the equilibrium point of system (1) is at ***O***(0, 0, 0, 0), indicating that this hyperchaotic system has only one equilibrium point, which is a unique characteristic of this system. The hyperchaotic system with a single equilibrium point offers significant advantages in both research and practical applications. Its streamlined system structure reduces complexity, thereby providing a clearer opportunity for dynamic behavior analysis and in-depth exploration in the research domain. This simplicity also facilitates the design of precise control strategies in practical applications, further enhancing system stability and controllability.

Linearizing system (1) at the equilibrium point ***O***(0, 0, 0, 0), the system’s Jacobian matrix, which is given by:

$${{\varvec{J}}}_{0}= \left( {\begin{array}{*{20}c}    { - {\text{a}}} & {\text{a}} & 0 & 1  \\    0 & {\text{c}} & 0 & 0  \\    0 & 0 & { - {\text{b}}} & 0  \\    0 & {\text{d}} & 0 & 0  \\   \end{array} } \right) $$

Let det(***J***_0_ − λ**I**), the eigenvalues of system (1) in the equilibrium point ***O***(0, 0, 0, 0) is:

$$ \lambda_{{1}} = - a,\lambda_{{2}} = c,\lambda_{{3}} = - b,\lambda_{{4}} = 0. $$

Since the parameters *a*, *b*, *c* are greater than 0, then the *λ*_2_ is a positive real number, *λ*_1_ and *λ*_3_ are negative real number. Hence, the equilibrium point is classified as a saddle point and is unstable. Therefore, the new hyperchaotic system (1) is a dissipative system. According to system equation, as known:

$$ \nabla {\mathbf{V}}{ = }\frac{{\partial \mathop {\text{x}}\limits^{.} }}{{\partial {\text{x}}}}{ + }\frac{{\partial \mathop {\text{y}}\limits^{.} }}{{\partial {\text{y}}}}{ + }\frac{{\partial \mathop {\text{z}}\limits^{.} }}{{\partial {\text{z}}}}{ + }\frac{{\partial \mathop {\text{w}}\limits^{.} }}{{\partial {\text{w}}}}{ = } - {\text{a + c}} - {\text{b = }} - {26} $$

When *t* → *∞*, each volume element of the trajectory of system (1) exponentially converges to zero with a rate of − 26, indicating that the trajectory of system (1) gradually moves towards a specific limit set of zero volume.

**Figure 1:**
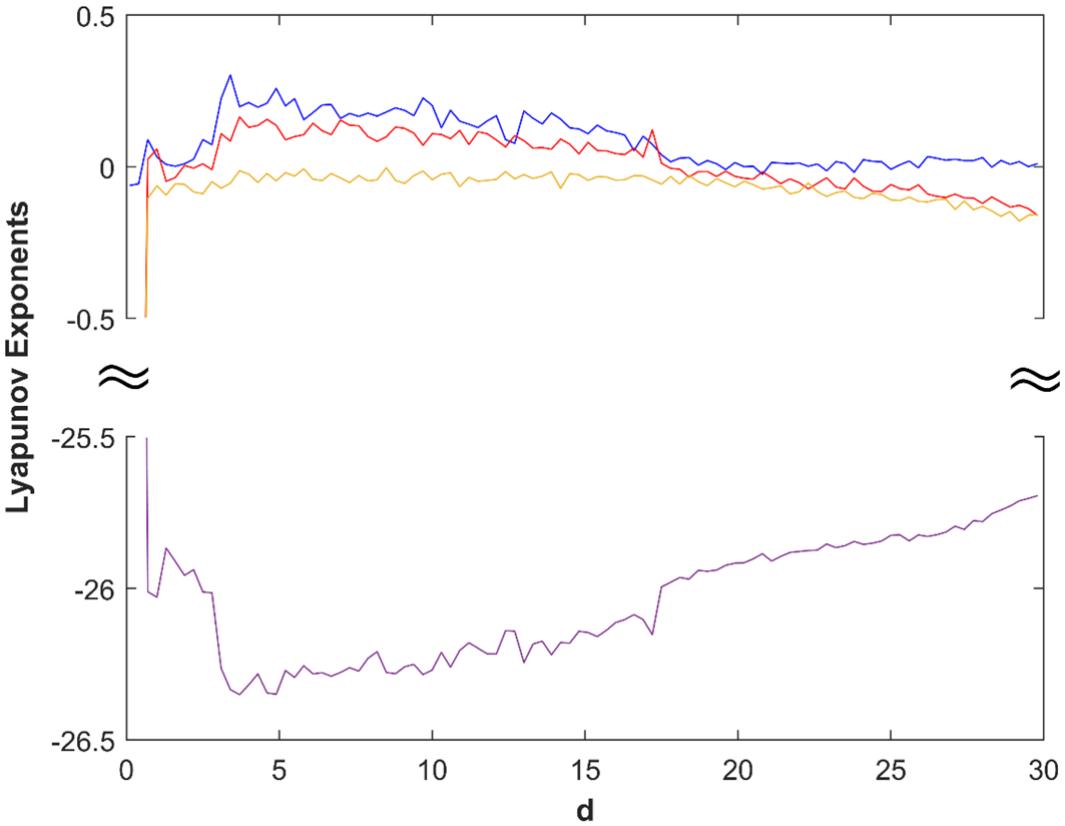
Lyapunov exponents spectrum of system (1).

**Figure 2:**
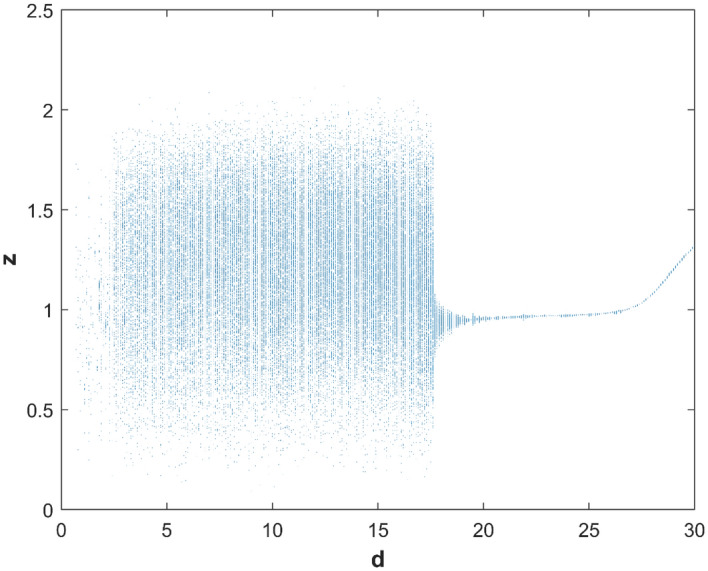
Bifurcation diagram of *z*.

Based on the numerical analysis results of Runge–Kutta method, the adjacent trajectories of the chaotic attractor in the state space exponentially separate from each other, which is a characteristic of chaos theory. The Lyapunov exponents are used to quantify the contraction or expansion of trajectories. Figure 1 shows the system's characteristic curve of the Lyapunov exponents as their change with parameter *d*. Additionally, the bifurcation of state variable *z* as it changes with *d* is shown in Fig. 2.

**Figure 3:**
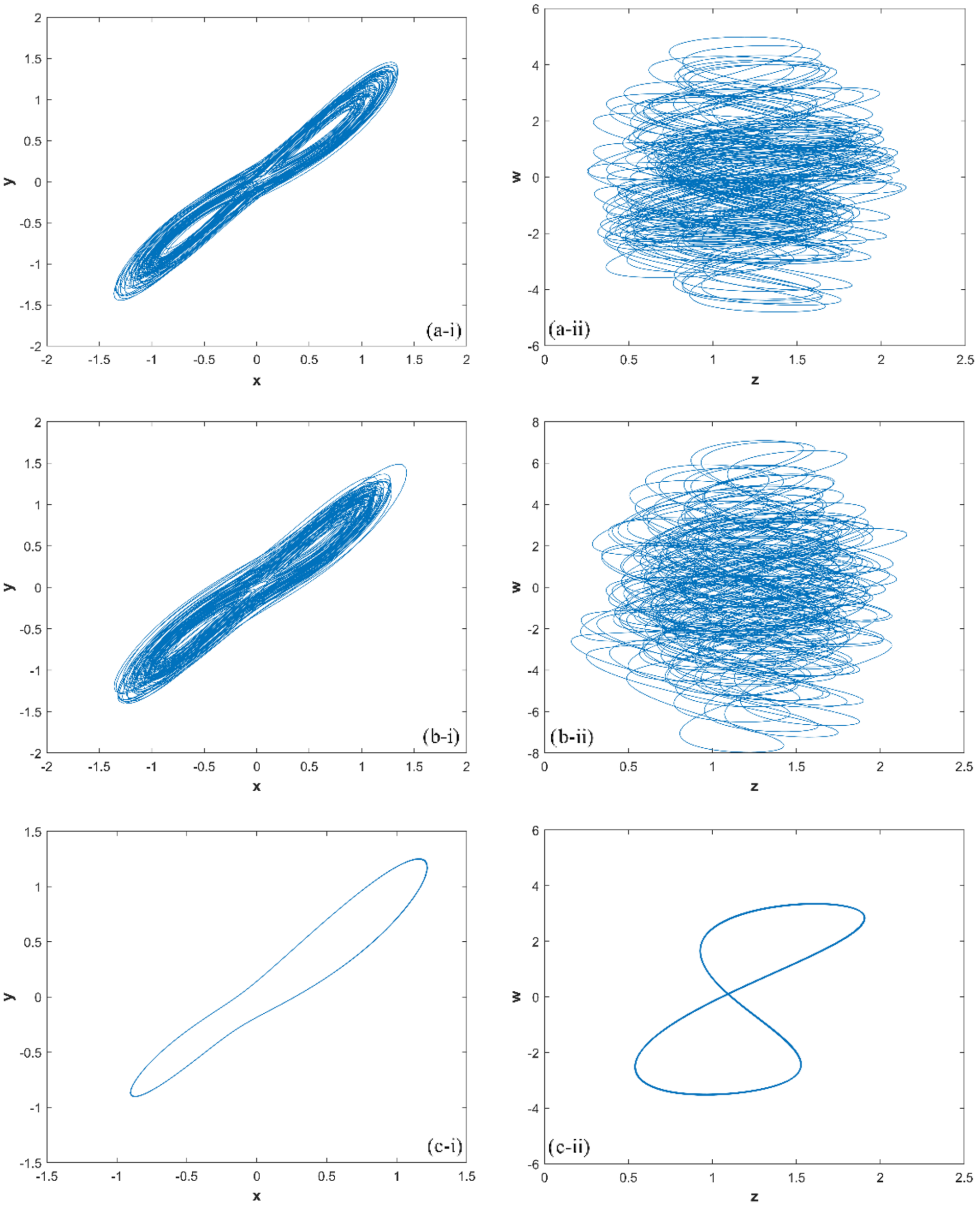
The hyperchaotic attractor of system (1) ((**a**) hyperchaotic attractor(*d* = 10); (**b**) chaotic attractor(*d* = 17); (**c**) periodic attractor (*d* = 22)).
- If 16 < *d* ≤ 18, there is one positive Lyapunov exponent, which indicates that system (1) is chaotic. When *d* = 17, the Lyapunov exponents of system (1) is (0.0932, 0, − 0.0336, − 26.1018), the chaotic attractor is shown in Fig. 3(b);
- If 18 < *d* ≤ 27, there are two zero Lyapunov exponents, which indicates that system (1) is quasi-periodic. When *d* = 22, the Lyapunov exponents of system (1) is (0, 0, − 0.0823, − 25.8783), the periodic orbit attractor is shown in Fig. 3c;
- If 27 < *d* ≤ 30, there is one zero Lyapunov exponent, and others are negative, which indicates that system (1) is stable at equilibrium ***O***(0, 0, 0, 0).

The match between the Lyapunov exponents spectrum and the bifurcation diagram confirms the existence of hyperchaotic states in the system. The changes in system (1) with parameter are described in detail as follows:

- If 0 < *d* ≤ 16, there are two positive Lyapunov exponents, which indicates that system (1) is hyperchaotic. When *d* = 10, the Lyapunov exponents of system (1) is (0.2027, 0.1091, 0, − 26.2692), the hyperchaotic attractor is shown in Fig. 3a;

## Feedback control of the hyperchaotic system

In the control of hyperchaotic systems, linear feedback control has more advantages compared to nonlinear control. Firstly, linear feedback controllers have simple mathematical forms, which are easy to design and implement. Secondly, for certain hyperchaotic systems, linear feedback controllers can achieve global stability of the system, while nonlinear controllers cannot guarantee system stability. Thirdly, linear feedback controllers have a certain robustness to parameter variations within a certain range, while nonlinear controllers may be too sensitive to parameter variations. Finally, the control strategy of linear feedback controllers is easy to interpret, which is beneficial for a deeper understanding of the control mechanism of the system. Therefore, utilizing linear feedback controllers to achieve feedback control of hyperchaotic systems is of greater research significance and value.

In this section, a linear feedback control term into the hyperchaotic system to stabilize it at the equilibrium point ***O***(0, 0, 0, 0). The controlled system of the hyperchaotic system (1) can be defined as:

2 $$ \left\{ \begin{gathered}   \frac{{{\text{dx}}}}{{{\text{dt}}}}\, =\,  - {\text{a}}\left( {{\text{y}} - {\text{x}}} \right){\text{ + w}} - {\text{k}}_{1} {\text{x}} \hfill \\   \frac{{{\text{dy}}}}{{{\text{dt}}}}\, =\, {\text{cy}} - 10{\text{xz}} - {\text{k}}_{2} {\text{y}} \hfill \\   \frac{{{\text{dz}}}}{{{\text{dt}}}}\, =\,  - {\text{bz + }}10{\text{xy}} - {\text{k}}_{3} {\text{z}} \hfill \\   \frac{{{\text{dw}}}}{{{\text{dt}}}}\, =\, {\text{dy + x}}^{2}  - {\text{k}}_{4} {\text{w}} \hfill \\  \end{gathered}  \right.. $$

The Jacobian matrix of the controlled system (2) can be obtained by linearizing around the equilibrium point ***O***(0, 0, 0, 0):

$$ {\text{J}}_{0}^{\prime } =\left( {\begin{array}{*{20}c}    { - {\text{a}} - {\text{k}}_{1} } & 0 & 0 & 1  \\    0 & {{\text{c}} - {\text{k}}_{2} } & 0 & 0  \\    0 & 0 & { - {\text{b}} - {\text{k}}_{3} } & 0  \\    0 & {\text{d}} & 0 & { - {\text{k}}_{4} }  \\   \end{array} } \right) $$

Let det(***J***_0_^’^ − *λ***I**) = 0, the eigenvalues of system (2) is calculated at the equilibrium point ***O***(0, 0, 0, 0) as:

$$ \lambda_{{1}} = - a - k_{{1}} ,\lambda_{{2}} = c - k_{{2}} ,\lambda_{{3}} = - b - k_{{3}} ,\lambda_{{4}} = - k_{{4}} . $$

**Figure 4:**
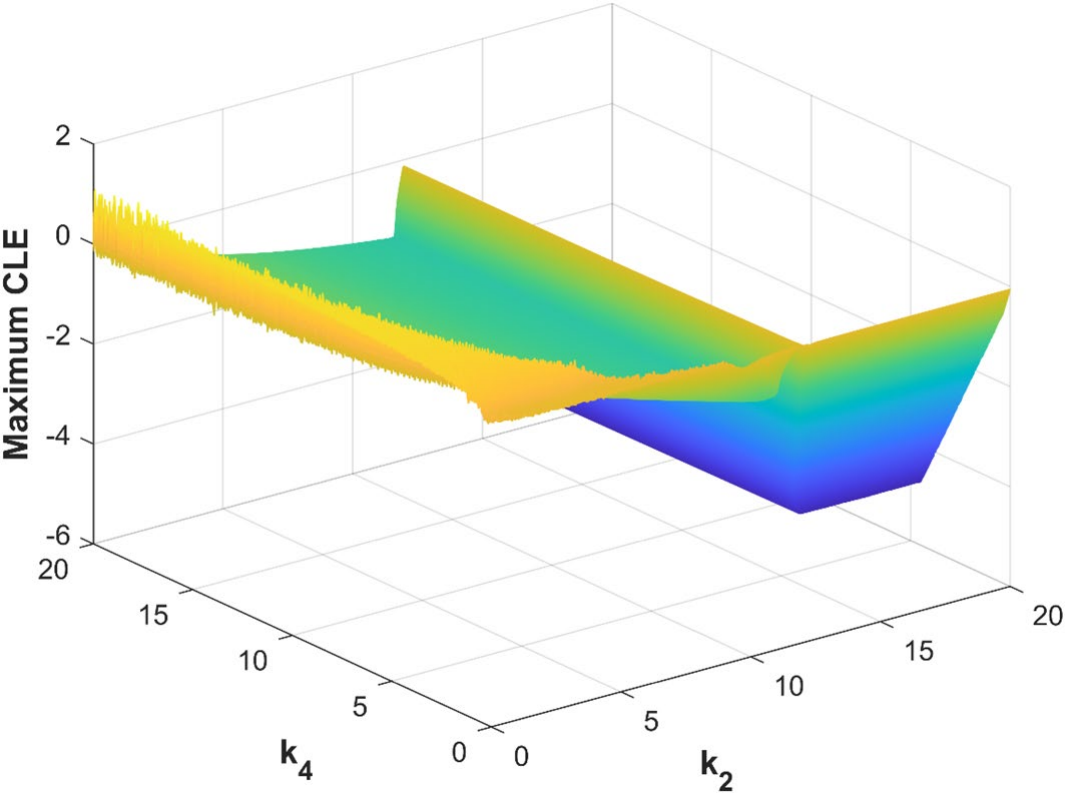
Maximum CLE of the controlled system (2) with k_2_ and k_4_.

If *a* + *k*_1_ > 0, *c*-*k*_2_ < 0, *b* + *k*_3_ > 0, *k*_4_ > 0, the *λ*_1_ < 0, *λ*_2_ < 0, *λ*_3_ < 0, *λ*_4_ < 0. Using the Routh‐Hurwitz theorem, the system would asymptotically stable to the equilibrium point ***O***(0,0,0,0) when there are only non-negative roots of controlled system (2). The *λ*_1_ = − *a*, *λ*_3_ = -*b*, and they are less than zero if *k*_1_ = 0, *k*_3_ = 0. Let *k*_2_ = 0–20, *k*_4_ = 0–20, the maximum Lyapunov exponents of system (2) change by control parameters *k*_2_, *k*_4_ are shown in Fig. 4. Figure 4 shows that the controlled system (2) would asymptotically stable to the equilibrium point ***O***(0, 0, 0, 0) from the experimental simulation of control parameters *k*_2_, *k*_4_, when *k*_2_ > 12, *k*_4_ > 0, here the *λ*_1_, *λ*_2_, *λ*_3_, *λ*_4_ are all negatives.

**Figure 5:**
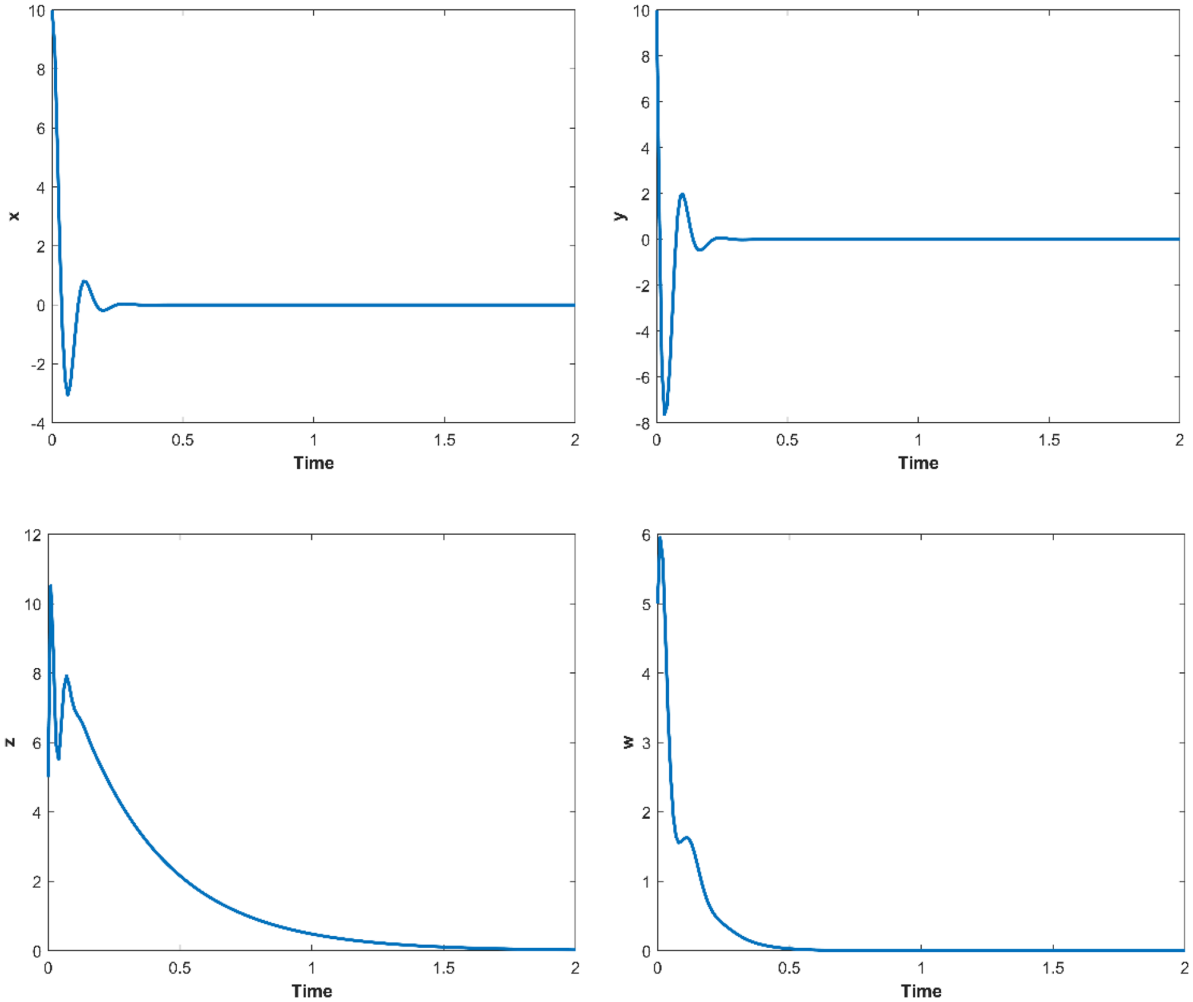
The Matlab trajectories of the variables of the controlled system (2).

When *a* = 35, *b* = 3, *c* = 12, *d* = 10, set the parameters as *k*_1_ = 0, *k*_2_ = 20, *k*_3_ = 0, *k*_4_ = 10, then *λ*_1_ = -35, *λ*_2_ = − 8, *λ*_3_ = − 3, *λ*_4_ = − 10, and the controlled system (2) would asymptotically stable to the equilibrium point ***O***(0, 0, 0, 0). When the initial values as (10, 10, 5, 5), the changes of the controlled system (2) with time are shown in Fig. 5. The Matlab simulation results show that the state variables in the controlled system (2) will gradually stabilize to the equilibrium point, and the time required from applying control to complete stability is less than 2 s.

## Multisim simulation results

The electronic circuit simulation software Multisim provides a highly visual simulation environment for hyperchaotic systems, allowing for the effortless creation and adjustment of circuit models and real-time observation of their behavior. This real-time feedback aids in a deeper understanding of the dynamic characteristics of hyperchaotic systems, laying the foundation for simulating the implementation of hyperchaotic systems in embedded hardware.

There are several software programs available for electronic circuit simulation, including PSPICE, LTSPICE, Multisim, and Simulink. In this paper, Multisim is chosen as the industry standard SPICE simulation and circuit design software for analog, digital, and power electronics in education and research.

**Figure 6:**
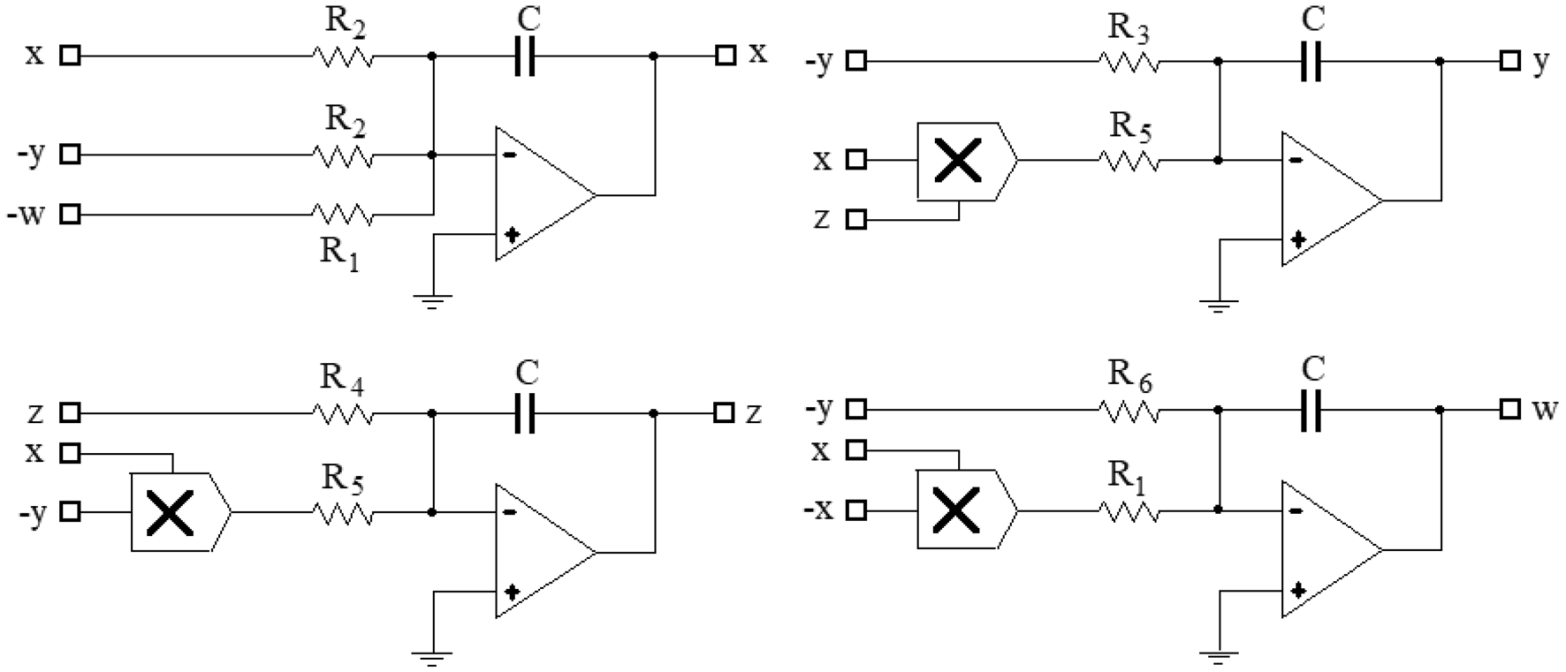
Circuit diagram for realizing hyperchaotic system (1).

The output voltages *v*_1_, *v*_2_, *v*_3_ and *v*_4_ of the circuit correspond to the four state variables *x*, *y*, *z* and *w*, respectively. To implement the hyperchaotic system (1), suitable components are selected, and the circuit diagram is shown in Fig. 6.

In this study, the circuit is composed of an operational amplifier (LF353N), a multiplier (AD633), linear resistors, and capacitors, with a power supply of ± 15 V. The operational amplifier is utilized for addition, subtraction, and integration of circuits, while the multiplier represents the nonlinearity of the system and connects the four state variables into a unified whole. The electronic circuit equations are obtained based on the hyperchaotic system (1) and are presented below:

3 $$ \left\{ \begin{gathered}   {\text{C}}\frac{{{\text{dv}}_{1} }}{{{\text{d}}\tau }}=\frac{{{\text{v}}_{2} }}{{{\text{R}}_{{11}} }} - \frac{{{\text{v}}_{1} }}{{{\text{R}}_{{12}} }}{\text{ + }}\frac{{{\text{v}}_{4} }}{{{\text{R}}_{{13}} }} \hfill \\   {\text{C}}\frac{{{\text{dv}}_{2} }}{{{\text{d}}\tau }}=\frac{{{\text{v}}_{2} }}{{{\text{R}}_{{21}} }} - \frac{{{\text{v}}_{1} {\text{v}}_{3} }}{{{\text{R}}_{{22}} }} \hfill \\   {\text{C}}\frac{{{\text{dv}}_{3} }}{{{\text{d}}\tau }}=\frac{{{\text{v}}_{3} }}{{{\text{R}}_{{31}} }}{\text{ + }}\frac{{{\text{v}}_{1} {\text{v}}_{2} }}{{{\text{R}}_{{32}} }} \hfill \\   {\text{C}}\frac{{{\text{d}}4}}{{{\text{d}}\tau }}=\frac{{{\text{v}}_{2} }}{{{\text{R}}_{{41}} }}{\text{ + }}\frac{{{\text{v}}_{1}^{2} }}{{{\text{R}}_{{42}} }} \hfill \\  \end{gathered}  \right.. $$

Let *R*_0_=100*k*Ω, *C*_0_=10*n*F, *t*_0_=*R*_0_*C*_0_, and *x*=*v*_1_, *y*=*v*_2_, *z*=*v*_3_, *w*=*v*_4_, *t*=*τ*/*t*, the electronic circuit Eq. (3) can be mapped as the dimensionless dynamic system as follows:

4 $$ \left\{ \begin{gathered}   \frac{{\text{C}}}{{{\text{C}}_{0} }}\frac{{{\text{dx}}}}{{{\text{d}}\tau }}=\frac{{{\text{R}}_{0} }}{{{\text{R}}_{{11}} }}{\text{y}} - \frac{{{\text{R}}_{0} }}{{{\text{R}}_{{12}} }}{\text{x + }}\frac{{{\text{R}}_{0} }}{{{\text{R}}_{{13}} }}{\text{w}} \hfill \\   \frac{{\text{C}}}{{{\text{C}}_{0} }}\frac{{{\text{dy}}}}{{{\text{d}}\tau }}=\frac{{{\text{R}}_{0} }}{{{\text{R}}_{{21}} }}{\text{y}} - \frac{{{\text{R}}_{0} }}{{{\text{R}}_{{22}} }}{\text{xz}} \hfill \\   \frac{{\text{C}}}{{{\text{C}}_{0} }}\frac{{{\text{dz}}}}{{{\text{d}}\tau }}= - \frac{{{\text{R}}_{0} }}{{{\text{R}}_{{31}} }}{\text{z + }}\frac{{{\text{R}}_{0} }}{{{\text{R}}_{{32}} }}xy \hfill \\   \frac{{\text{C}}}{{{\text{C}}_{0} }}\frac{{{\text{dw}}}}{{{\text{d}}\tau }}=\frac{{{\text{R}}_{0} }}{{{\text{R}}_{{41}} }}{\text{y + }}\frac{{{\text{R}}_{0} }}{{{\text{R}}_{{42}} }}{\text{x}}^{2}  \hfill \\  \end{gathered}  \right.. $$

Based on the dimensionless system (4), the circuit parameters are chosen as follows: resistor* R*_0_ = 100*k*Ω, capacitor* C*_0_ = 10*n*F, and time* t* = 1* ms*. Where capacitor* C* = 10*n*F, resistors *R*_13_ = *R*_42_ = *R*_1_ = 100*k*Ω, *R*_11_ = *R*_12_ = *R*_2_ = 2.86*k*Ω, *R*_21_ = *R*_3_ = 8.33*k*Ω, *R*_22_ = *R*_32_ = *R*_5_ = 10*k*Ω, *R*_31_ = *R*_4_ = 33.33*k*Ω, *R*_41_ = *R*_6_ = 3-20*k*Ω. *R*_6_ is adjusted using a potentiometer in Fig. 6 to obtain different values of parameter *d*.

**Figure 7:**
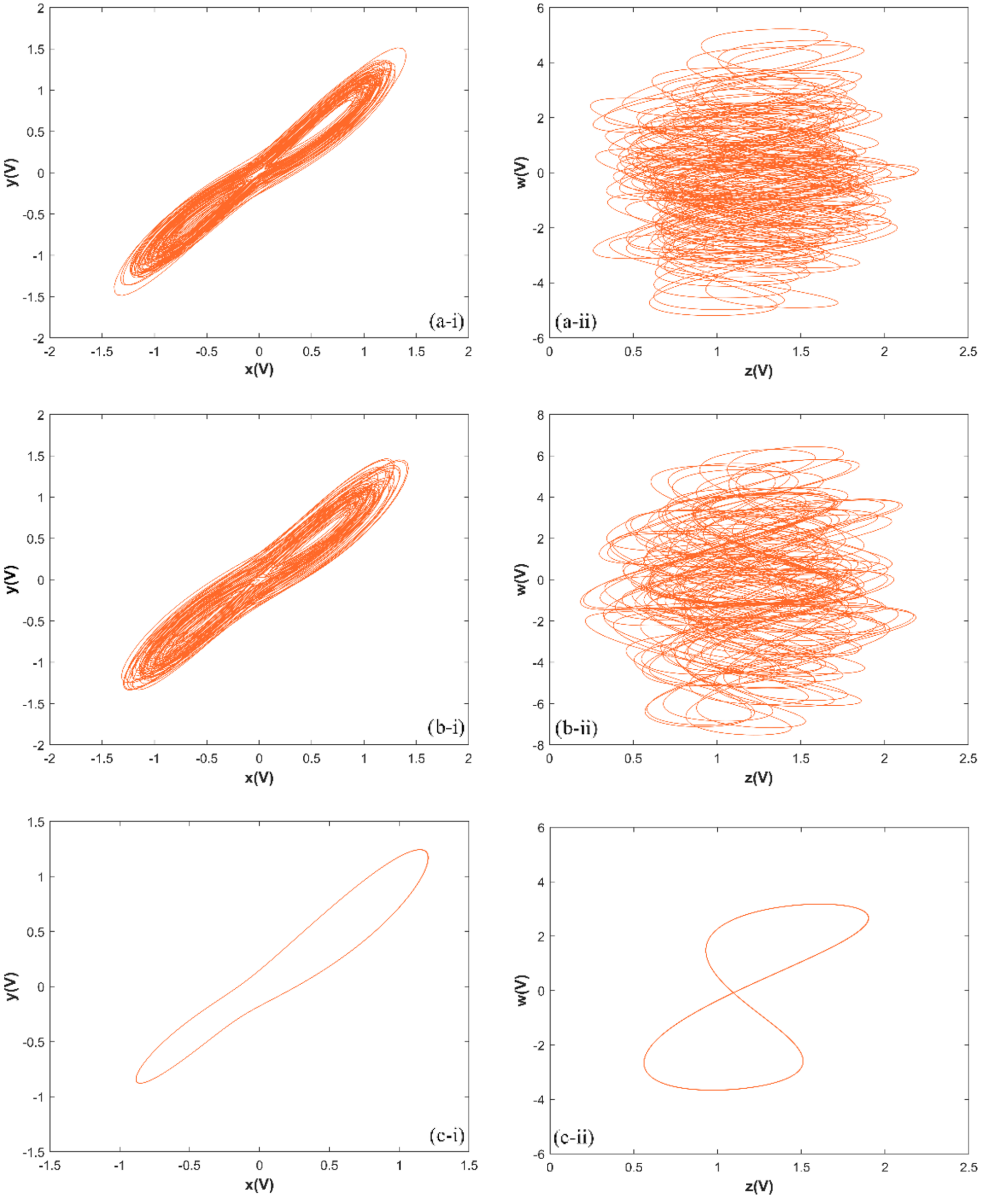
The phase portraits of system (3) simulated by Multisim ((**a**) hyperchaotic phase portraits (*R*_6_ = 10*k*Ω); (**b**) chaotic phase portraits ((*R*_6_ = 5.88*k*Ω); (**c**) periodic orbit phase portraits (*R*_6_ = 4.54*k*Ω)).

When *d* = 10, then *R*_6_ = 10*k*Ω, the phase transition trajectory of hyperchaotic signal is exhibited by an oscilloscope which is shown in Fig. 7a; When *d* = 17, the* R*_6_ = 5.88*k*Ω, the phase transition trajectory of chaotic signal is shown in Fig. 7b; When *d* = 22, the* R*_6_ = 4.54*k*Ω, the phase transition trajectory of periodic orbit is shown in Fig. 7c.

By utilizing the feedback control theory of the hyperchaotic system in conjunction with the controlled system (2), the electronic circuit Eq. (3), and the dimensionless dynamic system (4). When *a* = 35, *b* = 3, *c* = 12, *d* = 10, the dimensionless dynamic system of the controlled system as follows:

5 $$ \left\{ \begin{gathered}   \frac{{\text{C}}}{{{\text{C}}_{0} }}\frac{{{\text{dx}}}}{{{\text{d}}\tau }}=\frac{{{\text{R}}_{0} }}{{{\text{R}}_{{11}} }}{\text{y}} - \frac{{{\text{R}}_{0} }}{{{\text{R}}_{{12}} }}{\text{x + }}\frac{{{\text{R}}_{0} }}{{{\text{R}}_{{13}} }}{\text{w}} \hfill \\   \frac{{\text{C}}}{{{\text{C}}_{0} }}\frac{{{\text{dy}}}}{{{\text{d}}\tau }}=\frac{{{\text{R}}_{0} }}{{{\text{R}}_{{21}} }}{\text{y}} - \frac{{{\text{R}}_{0} }}{{{\text{R}}_{{22}} }}{\text{xz}} - \frac{{{\text{R}}_{0} }}{{{\text{R}}_{{23}} }}{\text{y}} \hfill \\   \frac{{\text{C}}}{{{\text{C}}_{0} }}\frac{{{\text{dz}}}}{{{\text{d}}\tau }}= - \frac{{{\text{R}}_{0} }}{{{\text{R}}_{{31}} }}{\text{z + }}\frac{{{\text{R}}_{0} }}{{{\text{R}}_{{32}} }}xy \hfill \\   \frac{{\text{C}}}{{{\text{C}}_{0} }}\frac{{{\text{dw}}}}{{{\text{d}}\tau }}=\frac{{{\text{R}}_{0} }}{{{\text{R}}_{{41}} }}{\text{y + }}\frac{{{\text{R}}_{0} }}{{{\text{R}}_{{42}} }}{\text{x}}^{2}  - \frac{{{\text{R}}_{0} }}{{{\text{R}}_{{43}} }}{\text{w}} \hfill \\  \end{gathered}  \right.. $$

**Figure 8:**
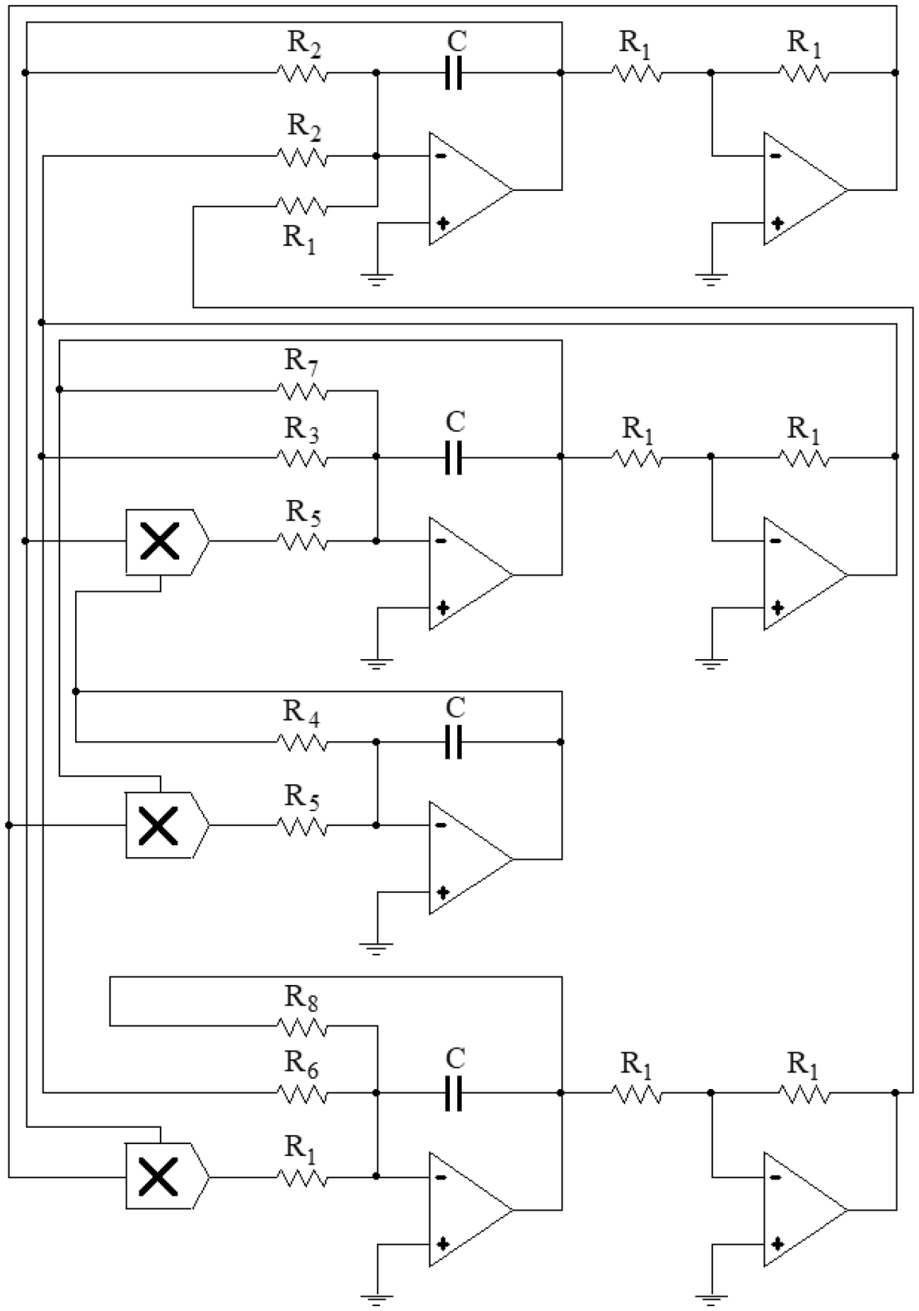
Schematic diagram of the controlled system (5).

The values of the electronic components used in the implementation of system (5) are given below: capacitor* C* = *C*_0_ = 10*n*F, resistor* R*_13_ = *R*_42_ = *R*_0_ = *R*_1_ = 100*k*Ω, *R*_11_ = *R*_12_ = *R*_2_ = 2.86*k*Ω, *R*_21_ = *R*_3_ = 8.33*k*Ω, *R*_22_ = *R*_32_ = *R*_41_ = *R*_43_ = *R*_5_ = *R*_6_ = *R*_8_ = 10*k*Ω, *R*_31_ = *R*_4_ = 33.33*k*Ω, *R*_23_ = *R*_7_ = 5*k*Ω. Figure 8 shows the Multisim circuit corresponding to the dimensionless power system (5) of the controlled system, contains 7 amplifiers (LF353N), 4 capacitors, 3 multipliers (AD633), and 17 resistors.

**Figure 9:**
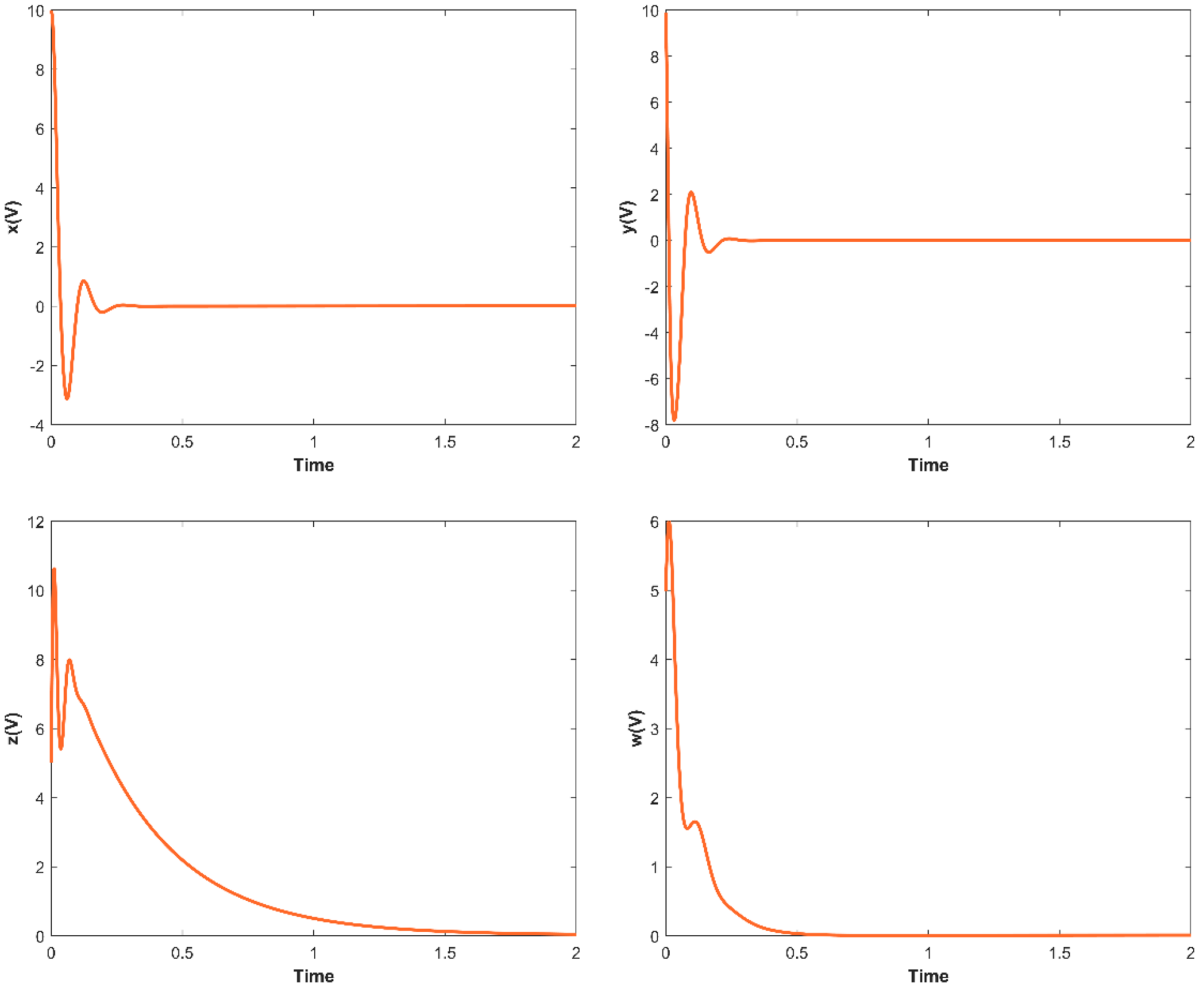
The Multisim trajectories of the variables of the controlled system (5).

The trajectories of the variables of the dimensionless power system (5) over time, as simulated in Multisim, are displayed in Fig. 9.

The consistency between the results obtained from the electronic circuit simulation software Multisim and the numerical simulation software Matlab provides robust validation for hyperchaotic attractors and linear feedback control of the new hyperchaotic system. Through a meticulous comparison of the output data from both software tools, a remarkably high degree of conformity in the dynamic behavior and evolution trends of attractors under varying conditions has been observed. Consequently, the consistency between numerical simulation results and electronic circuit simulation results not only reinforces the feasibility of the new hyperchaotic system but also furnishes reliable theoretical support for its practical application, establishing a strong foundation for the utilization of new hyperchaotic systems across various domains.

## Embedded hardware STM32 implementation results

The embedded hardware STM32 is a 32-bit microcontroller (MCU) based on ARM Cortex-M core, designed for low-power, high-performance, and low-cost embedded applications by STMicroelectronics. STM32 offers high performance, low power consumption, real-time capabilities, cost-effectiveness, and security. This is of great significance for applications requiring audio encryption, real-time signal processing, and complex mathematical computations, such as communication systems, audio storage devices, and speech recognition systems. In comparison to other platforms, it is better suited for applications demanding real-time audio encryption and complex mathematical calculations while maintaining low cost and high programmability.

In this paper, the STM32F103ZET6 is selected for implementing the phase diagram simulation and feedback control of the new hyperchaotic system. The STM32F103ZET operates at a frequency of 72 MHz and incorporates the high-performance ARM Cortex-M3 32-bit RISC core, high-speed embedded memories (Flash memory up to 512 Kbytes and SRAM up to 64 Kbytes), and a wide range of enhanced I/Os and peripherals connected to two APB buses. It has three 12-bit ADCs, one 12-bit DAC, four general-purpose 16-bit timers plus two PWM timers, and several standard and advanced communication interfaces, including two I2Cs, three SPIs, two I2Ss, one SDIO, five USARTs, a USB, and a CAN.

The embedded hardware results are displayed with an oscilloscope, which is Tektronix TBS1072C digital oscilloscope. It is a 2-channel digital storage oscilloscope that has a 70 MHz bandwidth and a sample rate of up to 1 GS/s. The oscilloscope also features a 7-inch WVGA color display that provides clear and detailed visualization of the signals. With these features, the Tektronix TBS1072C is an ideal tool for visualizing the signals generated by the embedded hardware during the experimentation process.

In experiment, the STM32 is used to verify the 4th-order Runge–Kutta method used for the discretization of the new hyperchaotic system (1), the iterative process is shown in Eq. (6). Where *a* = 35, *b* = 3, *c* = 12, *d* = 10, iteration step *h* = 0.05. $\tau$ represents the current moment, $x\left[\tau \right], y\left[\tau \right], z\left[\tau \right], w\left[\tau \right]$ are the values of the current moment, ${\text{temp\_x}}, \, {\text{temp\_y}}, \, {\text{temp\_z}}, \, {\text{temp\_w}}$ are the intermediate variables, ${\text{h}}_{1}{\text{\_x}}, \, {\text{h}}_{1}{\text{\_y}}, \, {\text{h}}_{1}{\text{\_z}}, \, {\text{h}}_{1}{\text{\_w}}$ are the slopes at the beginning of the time period, ${\text{h}}_{2}{\text{\_x}}, \, {\text{h}}_{2}{\text{\_y}}, \, {\text{h}}_{2}{\text{\_z}}, \, {\text{h}}_{2}{\text{\_w}}$ are the slopes of the midpoint of the time period, the slopes ${\text{h}}_{1}{\text{\_x}}, \, {\text{h}}_{1}{\text{\_y}}, \, {\text{h}}_{1}{\text{\_z}}, \, {\text{h}}_{1}{\text{\_w}}$ are used by Euler's method to determine the values of $x\left[\tau \right], y\left[\tau \right], z\left[\tau \right], w\left[\tau \right]$ at the point $\tau \text{+}h/2$, ${\text{h}}_{3}{\text{\_x}}, \, {\text{h}}_{3}{\text{\_y}}, \, {\text{h}}_{3}{\text{\_z}}, \, {\text{h}}_{3}{\text{\_w}}$ are the slopes of the midpoint, but this time the slopes ${\text{h}}_{2}{\text{\_x}}, \, {\text{h}}_{2}{\text{\_y}}, \, {\text{h}}_{2}{\text{\_z}}, \, {\text{h}}_{2}{\text{\_w}}$ are used to determine the $x\left[\tau \right], y\left[\tau \right], z\left[\tau \right], w\left[\tau \right]$ values, ${\text{h}}_{4}{\text{\_x}}, \, {\text{h}}_{4}{\text{\_y}}, \, {\text{h}}_{4}{\text{\_z}}, \, {\text{h}}_{4}{\text{\_w}}$ are the slopes of the end of the time period, and their $x\left[\tau \right], y\left[\tau \right], z\left[\tau \right], w\left[\tau \right]$ values are determined by ${\text{h}}_{3}{\text{\_x}}, \, {\text{h}}_{3}{\text{\_y}}, \, {\text{h}}_{3}{\text{\_z}}, \, {\text{h}}_{3}{\text{\_w}}$.

6 $$\left\{\begin{array}{c}{\text{h}}_{1}{\text{\_x}} \,= \, \left({\text{a}}\times \left({\text{y}}\left[\tau \right]-{\text{x}}\left[\tau \right]\right) + {\text{w}}\left[\tau \right]\right)\times h\\ {\text{h}}_{1}{\text{\_y}} \,= \, \left({\text{c}}\times {\text{y}}\left[\tau \right]\text{-10}\times {\text{x}}\left[\tau \right]\times {\text{z}}\left[\tau \right]\right)\times h\\ {\text{h}}_{1}{\text{\_z}} \,= \, \left(-{\text{b}}\times {\text{z}}\left[\tau \right]+ \text{10} \times {\text{x}}\left[\tau \right]\times {\text{y}}\left[\tau \right]\right)\times h\\ {\text{h}}_{1}{\text{\_w}} \,= \, \left({\text{d}}\times {\text{y}}\left[\tau \right] + {\text{x}}\left[\tau \right]\times {\text{x}}\left[\tau \right]\right)\times h\\ {\text{temp\_x}} \,= \, {\text{x}}\left[\tau \right] + {\text{h}}_{1}{\text{\_x}}/{2}\\ {\text{temp\_y}} \,= \, {\text{y}}\left[\tau \right] + {\text{h}}_{1}{\text{\_y}}/{2}\\ {\text{temp\_z}} \,= \, {\text{z}}\left[\tau \right] + {\text{h}}_{1}{\text{\_z}}/{2}\\ {\text{temp\_w}} \,= \, {\text{w}}\left[\tau \right] + {\text{h}}_{1}{\text{\_w}}/{2}\\ {\text{h}}_{2}{\text{\_x}} \,= \, \left({\text{a}}\times \left({\text{temp\_y}}-{\text{temp\_x}}\right) + {\text{temp\_w}}\right)\times h\\ {\text{h}}_{2}{\text{\_y}} \,= \, \left({\text{c}}\times {\text{temp\_y}}\text{-10}\times {\text{temp\_x}}\times {\text{temp\_z}}\right)\times h\\ {\text{h}}_{2}{\text{\_z}} \,= \, \left(-{\text{b}}\times {\text{temp\_z}}+ \text{10} \times {\text{temp\_x}}\times {\text{temp\_y}}\right)\times h\\ {\text{h}}_{2}{\text{\_w}} \,= \, \left({\text{d}}\times {\text{temp\_y}} + {\text{temp\_x}}\times {\text{temp\_x}}\right)\times h\\ {\text{temp\_x}} \,= \, {\text{x}}\left[\tau \right] + {\text{h}}_{2}{\text{\_x}}/{2}\\ {\text{temp\_y}} \,= \, {\text{y}}\left[\tau \right] + {\text{h}}_{2}{\text{\_y}}/{2}\\ {\text{temp\_z}} \,= \, {\text{z}}\left[\tau \right] + {\text{h}}_{2}{\text{\_z}}/{2}\\ {\text{temp\_w}} \,= \, {\text{w}}\left[\tau \right] + {\text{h}}_{2}{\text{\_w}}/{2}\\ {\text{h}}_{3}{\text{\_x}} \,= \, \left({\text{a}}\times \left({\text{temp\_y}}-{\text{temp\_x}}\right) + {\text{temp\_w}}\right)\times h\\ {\text{h}}_{3}{\text{\_y}} \,= \, \left({\text{c}}\times {\text{temp\_y}}\text{-10}\times {\text{temp\_x}}\times {\text{temp\_z}}\right)\times h\\ {\text{h}}_{3}{\text{\_z}} \,= \, \left(-{\text{b}}\times {\text{temp\_z}}+ \text{10} \times {\text{temp\_x}}\times {\text{temp\_y}}\right)\times h\\ {\text{h}}_{3}{\text{\_w}} \,= \, \left({\text{d}}\times {\text{temp\_y}} + {\text{temp\_x}}\times {\text{temp\_x}}\right)\times h\\ {\text{temp\_x}} \,= \, {\text{x}}\left[\tau \right] + {\text{h}}_{3}{\text{\_x}}\\ {\text{temp\_y}} \,= \, {\text{y}}\left[\tau \right] + {\text{h}}_{3}{\text{\_y}}\\ {\text{temp\_z}} \,= \, {\text{z}}\left[\tau \right] + {\text{h}}_{3}{\text{\_z}}\\ {\text{temp\_w}} \,= \, {\text{w}}\left[\tau \right] + {\text{h}}_{3}{\text{\_w}}\\ {\text{h}}_{4}{\text{\_x}} \,= \, \left({\text{a}}\times \left({\text{temp\_y}}-{\text{temp\_x}}\right) + {\text{temp\_w}}\right)\times h\\ {\text{h}}_{4}{\text{\_y}} \,= \, \left({\text{c}}\times {\text{temp\_y}}\text{-10}\times {\text{temp\_x}}\times {\text{temp\_z}}\right)\times h\\ {\text{h}}_{4}{\text{\_z}} \,= \, \left(-{\text{b}}\times {\text{temp\_z}}+ \text{10} \times {\text{temp\_x}}\times {\text{temp\_y}}\right)\times h\\ {\text{h}}_{4}{\text{\_w}} \,= \, \left({\text{d}}\times {\text{temp\_y}} + {\text{temp\_x}}\times {\text{temp\_x}}\right)\times h\\ x\left[\tau + \text{1} \right] \,= \, x\left[\tau \right] + \left({\text{h}}_{1}{\text{\_x}}+ \text{2} \times {\text{h}}_{2}{\text{\_x}}+ \text{2} \times {\text{h}}_{3}{\text{\_x}} + {\text{h}}_{4}{\text{\_x}}\right)/6\\ {\text{y}}\left[\tau + \text{1} \right] \,= \, {\text{y}}\left[\tau \right] + \left({\text{h}}_{1}{\text{\_y}}+ \text{2} \times {\text{h}}_{2}{\text{\_y}}+ \text{2} \times {\text{h}}_{3}{\text{\_y}} + {\text{h}}_{4}{\text{\_y}}\right)/6\\ {\text{z}}\left[\tau + \text{1} \right] \,= \, {\text{z}}\left[\tau \right] + \left({\text{h}}_{1}{\text{\_z}}+ \text{2} \times {\text{h}}_{2}{\text{\_z}}+ \text{2} \times {\text{h}}_{3}{\text{\_z}} + {\text{h}}_{4}{\text{\_z}}\right)/6\\ {\text{w}}\left[\tau + \text{1} \right] \,= \, {\text{w}}\left[\tau \right] + \left({\text{h}}_{1}{\text{\_w}}+ \text{2} \times {\text{h}}_{2}{\text{\_w}}+ \text{2} \times {\text{h}}_{3}{\text{\_w}} + {\text{h}}_{4}{\text{\_w}}\right)/6\end{array}\right.\text{.}$$

**Figure 10:**
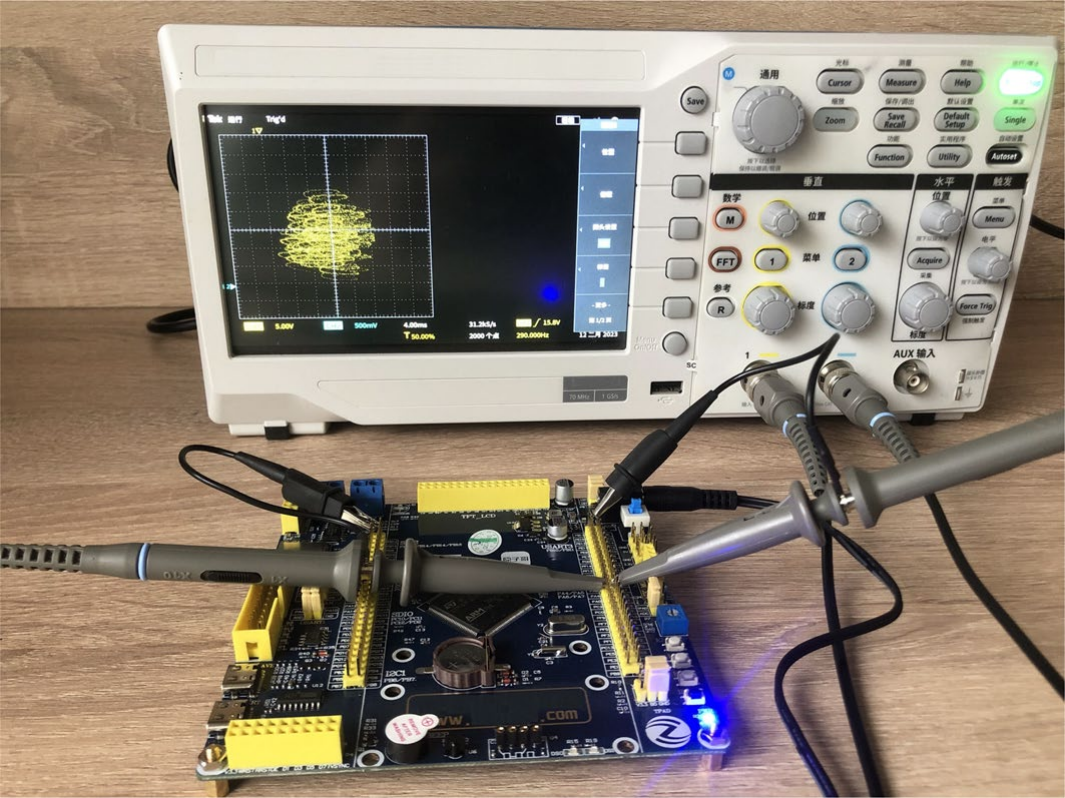
Experimental platform for STM32 implementation.

The STM32 generates a discrete hyperchaotic time series, and the DAC converter controls it through SPI to convert digital signals into analog signals, which are then sent to the oscilloscope for display. The platform of the embedded hardware STM32 is illustrated in Fig. 10.

**Figure 11:**
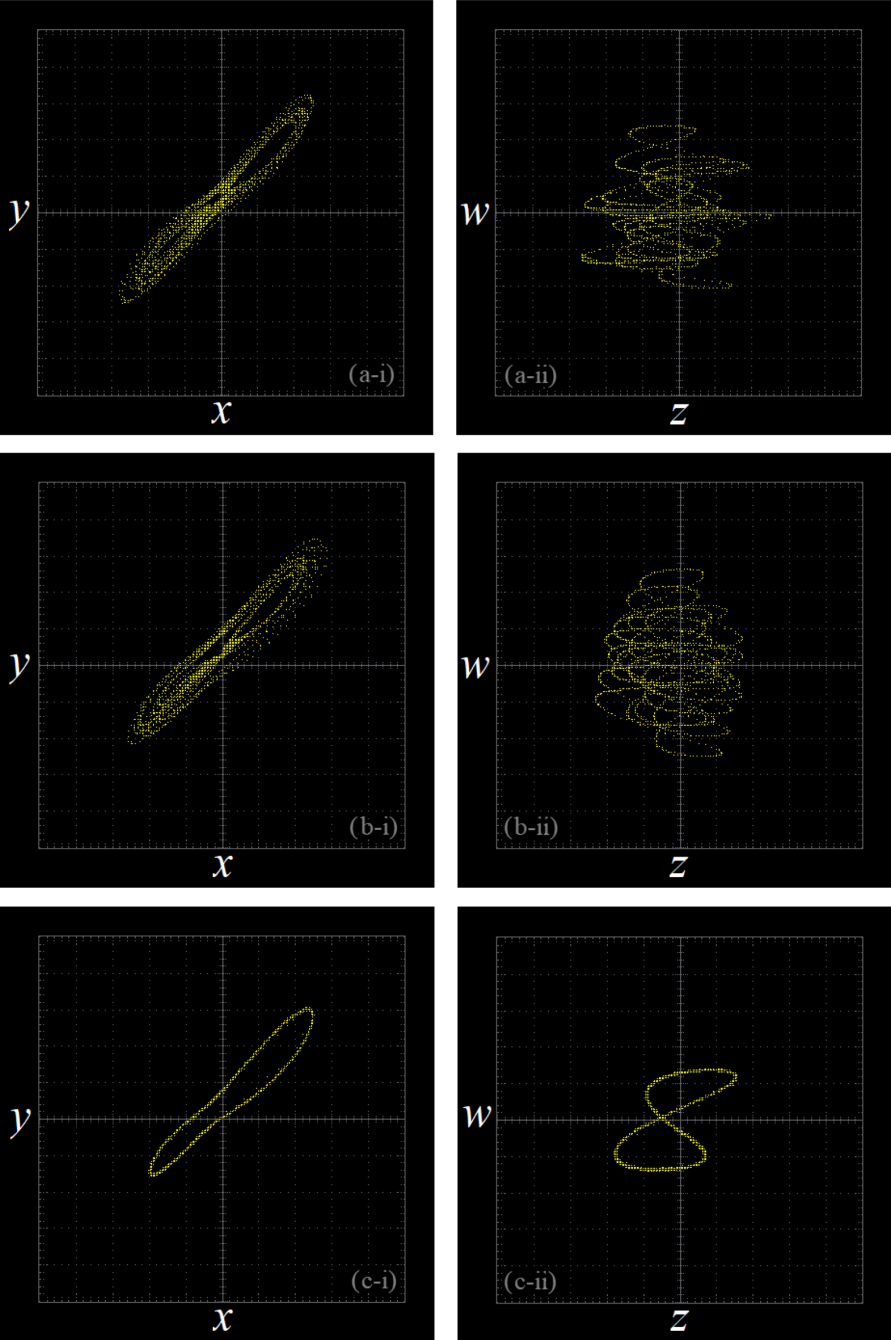
Experimental results of STM32 implementation ((**a**) hyperchaotic orbit (*d* = 10); (**b**) chaotic orbit (*d* = 17); (**c**) periodic orbit (*d* = 22)).

When *d* = 10, the phase transition trajectory of hyperchaotic signal is exhibited by an oscilloscope which is shown in Fig. 11a; When *d* = 17, the phase transition trajectory of chaotic signal is shown in Fig. 11b; When *d* = 22, the phase transition trajectory of periodic orbit is shown in Fig. 11c.

**Figure 12:**
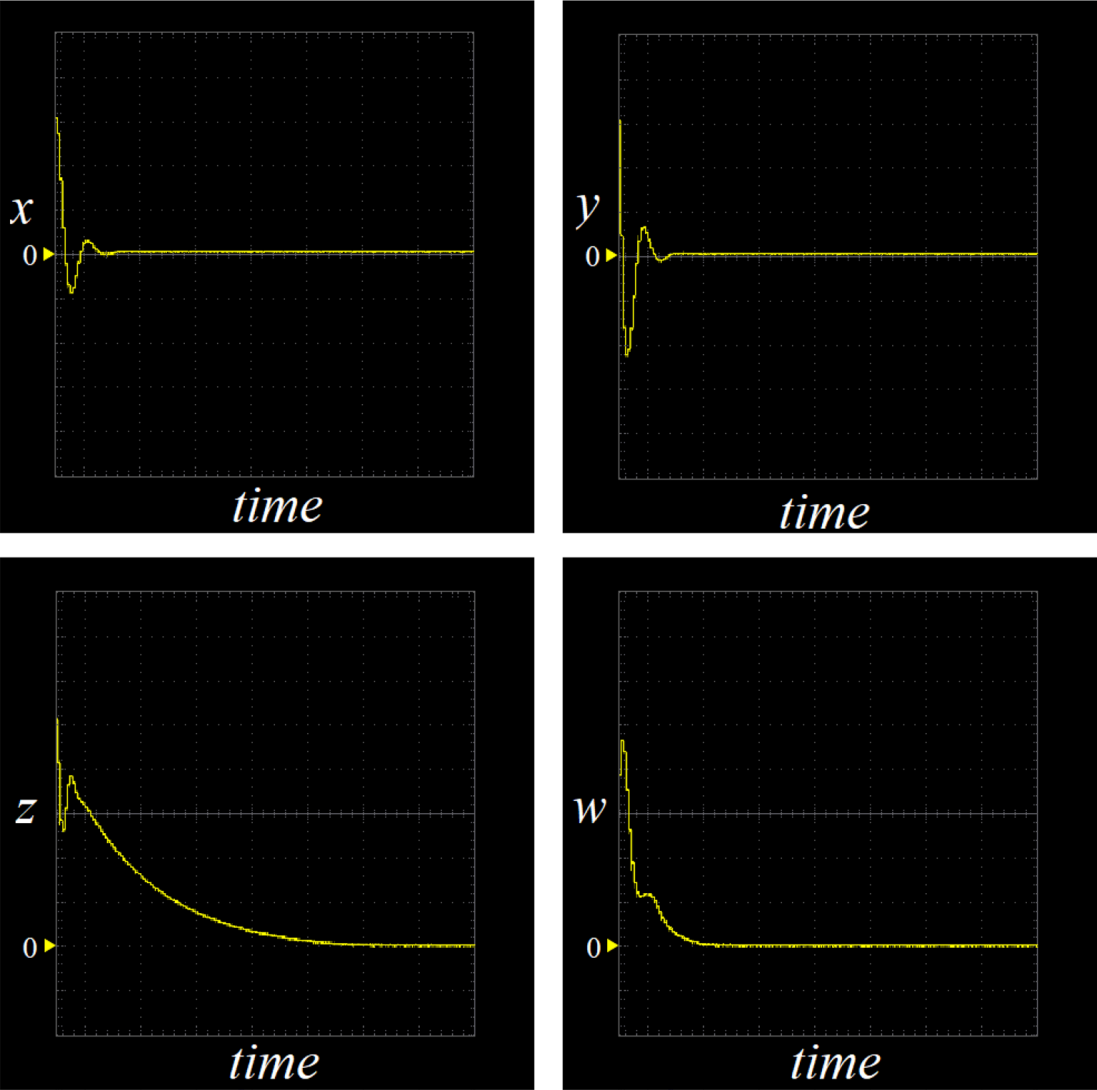
The trajectories of the variables of the controlled system (2) by STM32 implementation.

According to description of Sect. “Feedback control of the hyperchaotic system”, when *a* = 35, *b* = 3, *c* = 12, *d* = 10, let *k*_1_ = 0, *k*_2_ = 20, *k*_3_ = 0, *k*_4_ = 10, then *λ*_1_ = − 35, *λ*_2_ = − 8, *λ*_3_ = − 3, *λ*_4_ = − 10, the controlled system (2) would asymptotically stable to the equilibrium point ***O***(0, 0, 0, 0). If the initial values of controlled system (2) as (10,10,5,5), the changes of the controlled system (2) with parameter implemented by STM32 is shown in Fig. 12.

By comparing the results of Matlab numerical simulation in Fig. 5, the Multisim simulation in Fig. 9, and the STM32 implementation in Fig. 12, it can be seen that the results of all three methods are consistent. However, there may be some numerical accuracy loss due to the digital-to-analog and analog-to-digital conversion processes involved in the DAC output interface of STM32F103ZET and the digital oscilloscope, respectively. Nevertheless, the original characteristics of the hyperchaotic system are preserved.

## Audio encryption

In recent years, information security has become a global issue of concern. With the development of mobile internet, personal information, transaction information, and privacy information of netizens are all transmitted through the internet. Due to vulnerabilities in the network transmission process, user information leakage has become a significant security risk. Therefore, to solve this problem, it is necessary to use information encryption technology.

Confusion and diffusion are two common encryption techniques. Confusion typically refers to increasing the complexity of the ciphertext by using complex substitution rules or mixing the key with the plaintext. Diffusion involves ensuring that changes in individual plaintext bits propagate throughout the ciphertext so that even small changes in the plaintext result in significant differences in the ciphertext. In this paper, the audio encryption based on hyperchaotic sequences studied falls under the category of confusion methods.

Chaos encryption has become a research hotspot in information encryption due to its nonlinearity, real-time performance, security, anti-interference, and short key length. However, compared with chaotic encryption, hyperchaotic encryption has the following advantages:

- Higher security. Hyperchaotic systems have more complex dynamical behavior, and the output key sequence has higher randomness and unpredictability, thereby improving the security of the encryption system.
- Higher anti-interference. The key sequence output by the hyperchaotic system has a higher range and frequency of disturbance, which can effectively resist common attack methods such as noise attacks and interference attacks.
- Better scalability. Hyperchaotic systems can achieve high-dimensional expansion, that is, multiple chaotic systems' functions can be implemented in a hyperchaotic system, making it applicable to more complex encryption systems.
- Stronger self-synchronization. The key sequence output by the hyperchaotic system can automatically synchronize during the encryption and decryption process without external synchronization control, making the encryption system simpler and more practical.

In this section, the audio encryption algorithm based on the hyperchaotic key sequence is studied, using the 4D hyperchaotic system that has been constructed. Then designed a method to generate hyperchaotic key sequence, and implemented audio encryption using the cross-XOR operation. The experiment was conducted and validated on the embedded hardware STM32.

### Hyperchaotic key sequence

Due to the high randomness and complexity of the hyperchaotic system, the security of the encryption algorithm is enhanced. The generation of hyperchaotic key sequence transforms the hyperchaotic sequence into the required form of the key sequence through a specific quantization algorithm. The steps are as follows:

**Step 1:** Pre-iterate the hyperchaotic system ***N***_1_ times to eliminate the transient effects of the hyperchaotic system entering a hyperchaotic state.

**Step 2:** Iterate the hyperchaotic system ***N***_2_ times to generate a new set of state values ***A*** = {***A****x*, ***A****y*, ***A****z*, ***A****w*}, where ***A****x* = {*x*_1_, *x*_2_, *x*_3_, …, *x*_k_}, ***A****y* = {*y*_1_, *y*_2_, *y*_3_, …, *y*_k_}, ***A****z* = {*z*_1_, *z*_2_, *z*_3_, …, *z*_k_}, and ***A****w* = {*w*_1_, *w*_2_, *w*_3_, …, *w*_k_}, 0 < *k* ≤ ***N***_2_. Then, scale down the state values ***A*** down to 1/*p* and take *q* decimal places to generate the new state values ***B*** = {***B****x*, ***B****y*, ***B****z*, ***B****w*}, where ***B****x* = {*bx*_1_, *bx*_2_, *bx*_3_, …, *bx*_k_}, ***B****y* = {*by*_1_, *by*_2_, *by*_3_, …, *by*_k_}, ***B****z* = {*bz*_1_, *bz*_2_, *bz*_3_, …, *bz*_k_}, and ***B****w* = {*bw*_1_, *bw*_2_, *bw*_3_, …, *bw*_k_}. The conversion formula is as follows:

$$\left\{\begin{array}{c}B=A*{10}^{q-p}-floor\left({\varvec{A}}*{10}^{q-p}\right),where \,A\ge 0,\\ B=floor\left({\varvec{A}}*{10}^{q-p}\right)-A*{10}^{q-p},where\, A<0\end{array}\right.$$

where *floor*(∙) denotes the floor function, and *p* and *q* are adjustable parameters, where *p* represents scaling down the value by 1/*p*, and *q* represents taking *q* decimal places as the new state value.

**Step 3:** Calculate the key sequence. According to the positive and negative changes in the numerical value of the state value ***A***, adjust the order of the state value ***B*** to generate the key sequence ***K*** = {***K***_1_, ***K***_2_, ***K***_3_, …, ***K***_k_}. The mapping relationship between the sorting rule of state value ***A***, ***B*** and the generation of key sequence K is shown in Table 1.

**Table 1:** The mapping relationship between the sorting rule of state value ***A***, ***B*** and the generation of key sequence ***K***.

State values ***A***				Key sequence ***K***_k_
*x*	*y*	*z*	*w*	
+	+	+	+	*bx*_k_*,by*_k_*,bz*_k_*,bw*_k_
+	+	+	−	*bx*_k_*,by*_k_*,bw*_k_*,bz*_k_
+	+	−	+	*bx*_k_*,bz*_k_*,by*_k_*,bw*_k_
+	+	−	−	*bx*_k_*,bz*_k_*,bw*_k_*,by*_k_
+	−	+	+	*by*_k_*,bx*_k_*,bz*_k_*,bw*_k_
+	−	+	−	*by*_k_*,bx*_k_*,bw*_k_*,bz*_k_
+	−	−	+	*by*_k_*,bz*_k_*,bx*_k_*,bw*_k_
+	−	−	−	*by*_k_*,bz*_k_*,bw*_k_*,bx*_k_
−	+	+	+	*bz*_k_*,bx*_k_*,by*_k_*,bw*_k_
−	+	+	−	*bz*_k_*,bx*_k_*,bw*_k_*,by*_k_
−	+	−	+	*bz*_k_*,by*_k_*,bx*_k_*,bw*_k_
−	+	−	−	*bz*_k_*,by*_k_*,bw*_k_*,bx*_k_
−	−	+	+	*bw*_k_*,bx*_k_*,by*_k_*,bz*_k_
−	−	+	−	*bw*_k_*,bx*_k_*,bz*_k_*,by*_k_
−	−	−	+	*bw*_k_*,by*_k_*,bx*_k_*,bz*_k_
−	−	−	−	*bw*_k_*,by*_k_*,bz*_k_*,bx*_k_

**Step 4:** Repeat steps 2 and 3 for (Frame_Len/4–1) times to generate a key matrix ***K*** with a length of (Frame_Len*4), where Frame_Len is the length of one frame of audio data.

### Cross-XOR operation

To demonstrate the simplicity and non-linearity advantages of implementing super-chaotic keys, and to fully utilize the advantages of ciphertext interleaving diffusion technology in audio encryption and improve its ability to resist illegal attacks, a new method of cross-XOR operation is introduced. Its features are suitable for audio signal encryption, non-linear ciphertext, easy to implement, can improve the speed of ciphertext diffusion.

**Figure 13:**
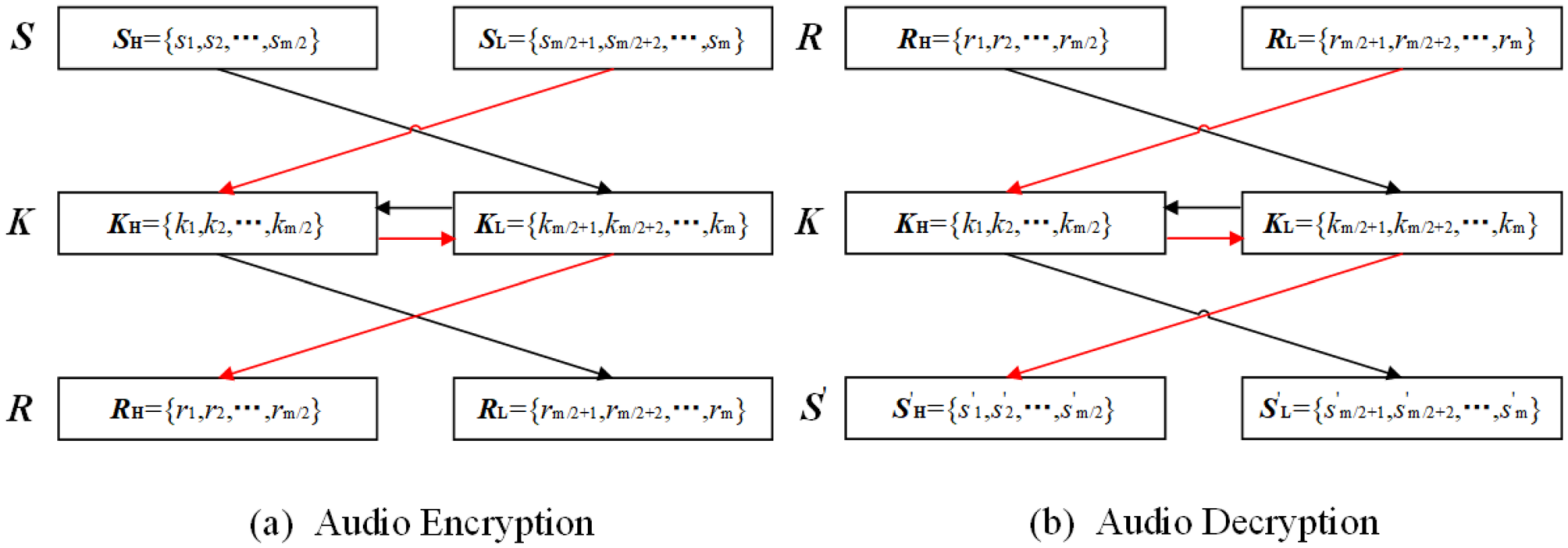
Block diagrams of cross-XOR operation and its audio encryption and decryption processes.

Assuming that the audio sequence ***S*** and key sequence ***K*** are m-bit binary sequences, that is, ***S*** = {*s*_1_,*s*_2_,*s*_3_,…,*s*_m_}, ***K*** = {*k*_1_,*k*_2_,*k*_3_,…,*k*_m_}, ***S***_H_ and ***S***_L_ represent the high *m*/2-bit and low *m*/2-bit sub-sequences of the audio sequence ***S***, and ***K***_H_ and ***K***_L_ represent the high *m*/2-bit and low *m*/2-bit sub-sequences of the key sequence ***K***. The implementation of audio encryption based on cross-XOR operation is shown in the following equation:

$$ {\varvec{R}}_{{\text{H}}} = {\varvec{S}}_{{\text{L}}} \oplus {\varvec{K}}_{{\text{H}}} \oplus {\varvec{K}}_{{\text{L}}} ,{\varvec{R}}_{{\text{L}}} = {\varvec{S}}_{{\text{H}}} \oplus {\varvec{K}}_{{\text{H}}} \oplus {\varvec{K}}_{{\text{L}}} ,{\varvec{R}} = \, \left( {{\varvec{R}}_{{\text{H}}} ,{\varvec{R}}_{{\text{L}}} } \right), $$

where ***R*** sequence is the result of audio encryption using cross-XOR operation, where ***R***_H_ and ***R***_L_ represent the high *m*/2-bit and low *m*/2-bit sub-sequences of the key sequence ***R***. The audio decryption is the inverse operation of encryption, which is implemented as shown in the following equation:

$$ {\varvec{S}}_{{\text{H}}}^{\prime } = {\varvec{R}}_{{\text{L}}} \oplus {\varvec{K}}_{{\text{H}}} \oplus {\varvec{K}}_{{\text{L}}} ,{\varvec{S}}_{{\text{L}}}^{\prime } = {\varvec{R}}_{{\text{H}}} \oplus {\varvec{K}}_{{\text{H}}} \oplus {\varvec{K}}_{{\text{L}}} ,S^{\prime } = \, \left( {{\varvec{S}}_{{\text{H}}}^{\prime } ,{\varvec{S}}_{{\text{L}}}^{\prime } } \right), $$

where S' sequence is the result of audio decryption using cross-XOR operation, where ***S'***_H_ and ***S'***_L_ represent the high *m*/2-bit and low *m*/2-bit sub-sequences of the key sequence ***S'***. The audio encryption and decryption processes based on cross-XOR operation are shown in Fig. 13.

## Experimental results

**Figure 14:**
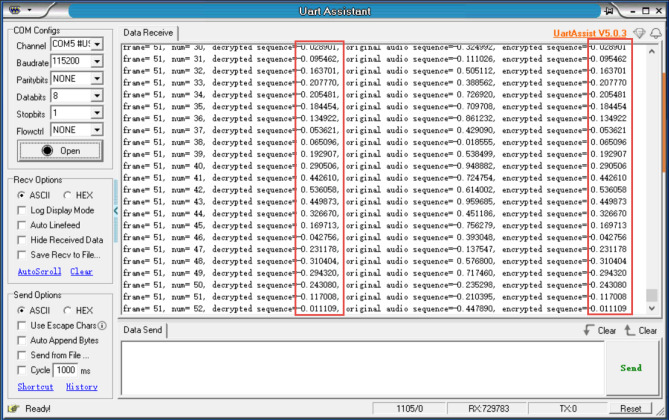
The encrypted and decrypted sequences printed by the serial port debugging tool.

**Figure 15:**
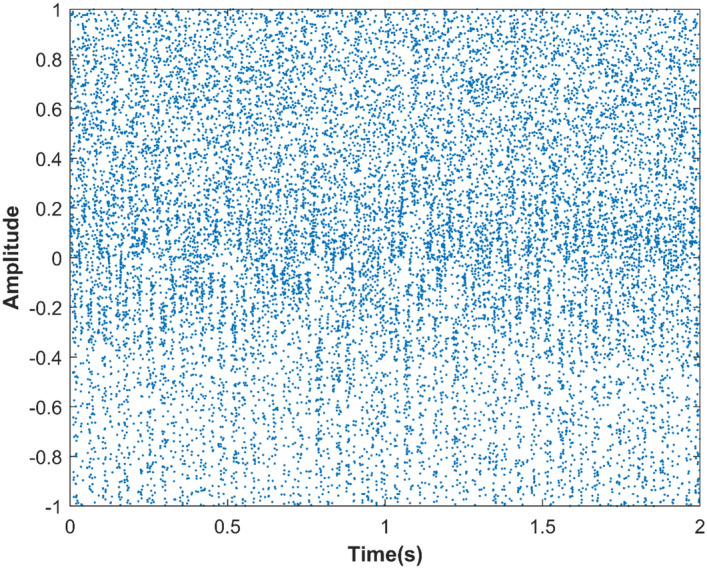
Hyperchaotic key sequence.

In this experiment, the embedded hardware STM32F103ZET6 was used to implement audio encryption based on the hyperchaotic key sequence. Since the embedded hardware STM32F103ZET6 does not integrate an audio acquisition module and display, the data input is imported into the embedded hardware STM32 in the form of a list. The output of the encrypted and decrypted audio sequences is exported to the PC through the serial port of the embedded hardware STM32F103ZET6, and then printed out using a serial port debugging tool, as shown in Fig. 14. Figure 15 is the hyperchaotic key sequence.

**Figure 16:**
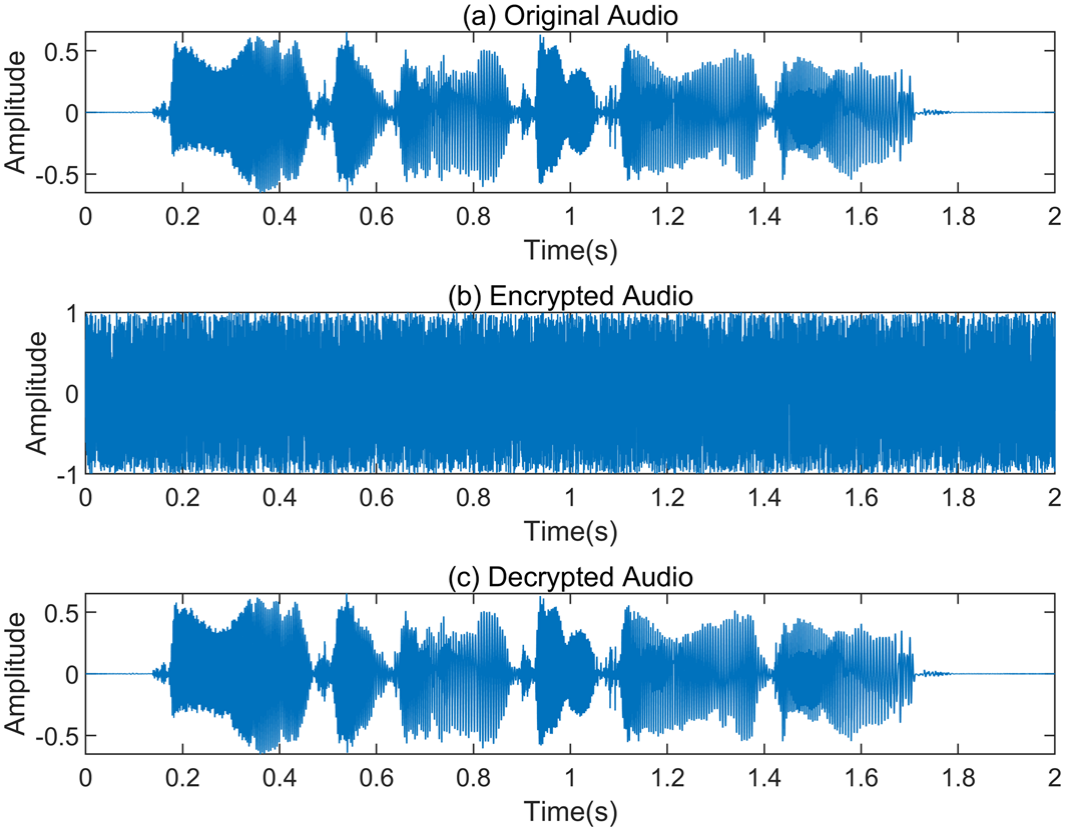
Time domain data before and after audio encryption.

**Figure 17:**
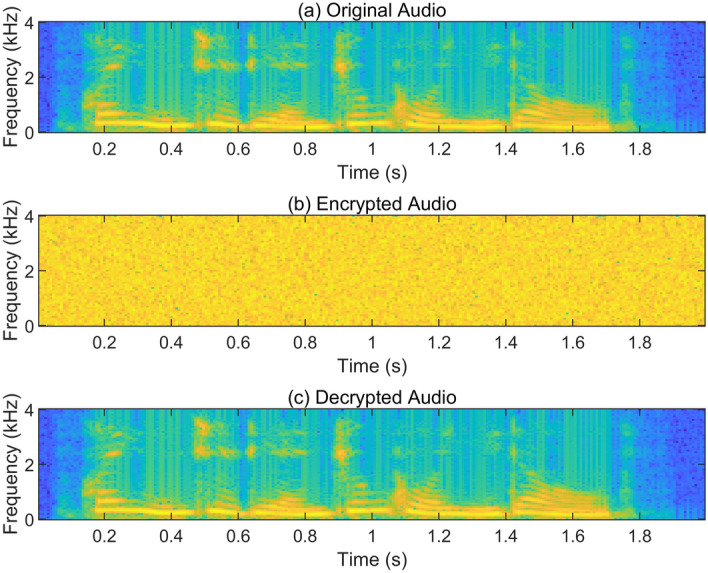
Spectrograms of the audio sequence before and after encryption.

The data sequence output by the embedded hardware STM32F103ZET6 serial port is transmitted to the PC through the serial port, and the audio sequence is displayed using Matlab, as shown in Figs. 16 and 17.

Figure 16a shows the original audio data in the time domain before encryption, Fig. 16b shows the encrypted audio data in the time domain, and Fig. 16c shows the decrypted audio data in the time domain. By comparing Fig. 16a and c, it shows that the time domain data of the audio sequence before and after encryption is identical, which indicates that the hyperchaotic key sequence did not cause distortion to the audio data. At the same time, from Fig. 16b, it shows that the time domain distribution of the audio sequence after encryption with the hyperchaotic key sequence is completely chaotic and different from the time domain pattern of the original audio data. This demonstrates that the encrypted ciphertext has completely concealed the characteristic information of the original audio data.

Figure 17 shows the spectrograms of the audio signal before encryption, after encryption, and after decryption. In Fig. 17b, it is evident that the encrypted audio signal exhibits a wide frequency distribution, resembling random noise in the time–frequency domain. Comparing Fig. 17a and c, the frequency domain information remains consistent before and after audio signal encryption. This implies that the decrypted audio signal maintains its original characteristics without any distortion, confirming the effectiveness of the hyperchaotic sequence encryption.

## Conclusions

In this paper, a new 4D hyperchaotic system is proposed, and its basic dynamic behavior is analyzed, including its chaotic attractor, equilibrium point stability, Lyapunov exponent spectrum, bifurcation diagram, and so on. The study demonstrates that the system has only one equilibrium point and remains hyperchaotic by varying its parameters over a wide range. A linear feedback control approach is introduced to stabilize the hyperchaotic system to its equilibrium point, and the effectiveness of this approach is proven through both Multisim circuit simulation and embedded hardware STM32 implementation.

Furthermore, the designed hyperchaotic system is applied to audio encryption in this paper. The audio encryption algorithm employs the cross-XOR operation method, which is simple to implement and has a certain degree of complexity. The experimental results show that the encrypted audio sequence is identical to the original sequence in the time domain, while it appears as random noise in the frequency domain, making it impossible to discern the actual information carried by the audio. This proves the effectiveness of the cross-XOR operation method based on hyperchaotic key sequence for audio encryption.

In future work, the high complexity of the hyperchaotic key sequence will be leveraged to study the master–slave mode of data encryption and decryption using embedded hardware STM32, which has significant research value in areas such as image encryption, audio encryption, video encryption, and more.

## Data Availability

The data used to support the findings of this study are available from the corresponding author upon request.
